# Brain Tumors in Saudi Arabia: An Observational and Descriptive Epidemiological Analysis

**DOI:** 10.3390/healthcare10091796

**Published:** 2022-09-18

**Authors:** Ahmad Almatroudi

**Affiliations:** Department of Medical Laboratories, College of Applied Medical Sciences, Qassim University, Buraydah 52571, Saudi Arabia; aamtrody@qu.edu.sa

**Keywords:** brain tumor, ASIR (age-standardized incidence rate), CIR (crude incidence rate), Saudi Arabia

## Abstract

Introduction: Brain tumors are one of the major causes of death and morbidity around the world. A prospective existential retrospective observational population-cohort study based on the comprehensive research work on brain tumors in the Saudi population was conducted, with statistics drawn from the Saudi Cancer Registry data collected and published by the Ministry of Health, Saudi Arabia, which is a national document prepared and maintained by the National Health Information Center, from 2006 to 2016. For the analysis of the brain tumor distribution and trends in Saudi Arabian inhabitants, the current study outlined the brain tumor incident rates in the age-standardized incidence rates (ASIRs) and crude incidence rates (CIRs) in the inhabitants of Saudi Arabia by distinct age cohorts, the year of diagnosis, and the core administrative regions of Saudi Arabia. Method: Statistical tools, such as GraphPad Prism and SPSS 2.0, were used for the analyses of the *t*-test, Kruskal–Wallis test, and descriptive statistics, including the sex ratio and other demographic features. Between 2006 and 2016, Saudi Arabia recorded 1854 and 1293 cases of brain tumors in males and females, respectively. Results: The highest percentage and mean number of brain tumor cases were recorded among males and females in the age group 0–4 years, and the lowest proportion of brain tumor cases were reported among males and females in the higher age group (55–69 years). The highest mean CIR and ASIR were found in the male and female populations of the Riyadh region, and the highest CIR and ASIR sex ratios were found in the Baha and Naj regions of Saudi Arabia, respectively. Males in the Jazan region had the lowest average CIRs and ASIRs. The Baha and Jazan regions of Saudi Arabia recorded the lowest mean CIR and ASIR among females. Conclusion: The Riyadh region had the most significant increases in ASIRs and CIRs for brain tumors in males and females from 2006 to 2016, whereas the Jazan region had the least significant changes in the ASIRs in males and females.

## 1. Introduction

Cancer is one of the major causes of mortality worldwide, and it is a significant barrier to increasing life expectancy. As per the World Health Organization (WHO) report published in 2019 [1], cancer is the first or second major cause of mortality in 112 nations, and the third or fourth in another 23 countries [2]. According to the International Agency for Research on Cancer (IARC), the number of new cancer diagnoses increased to over 18 million in 2018, with approximately 9 million cancer deaths. Brain and other nervous system cancers are the tenth leading cause of death for men and women. Brain tumors are one of the common causes of the deaths worldwide, and they account for about 3% of the mortality of all the cancers’ allied deaths. Worldwide, an estimated 251,329 people died from primary brain cancer and CNS (central nervous system) tumors in 2020. It is also known as an intracranial tumor, and it is divided into two groups: one is primary (originates from the tissue of the brain), further categorized as glial (composed of glial cells) or nonglial (developed on or in the structures of the brain, including nerves, blood vessels, and glands), and benign or malignant; the second is metastatic (originates from another part of the body and migrates to the brain). There are more than 150 types of brain tumors. Despite their rarity, brain tumors are a considerable cause of death, and particularly in children and young adults, where they account for roughly 30% and 20% of cancer fatalities, respectively. In comparison with other malignancies, they also produce a high rate of death. In 2015, brain tumors were expected to account for 1.4 percent of all new cancer diagnoses, and 2.6 percent of all cancer deaths [3]. Chordomas, craniopharyngiomas, gangliocytomas, glomus jugulare tumors, meningiomas, pineocytomas, pituitary adenomas, Schwannomas, gliomas, astrocytomas, ependymomas, glioblastoma multiforme (GBM), medulloblastomas, oligodendrogliomas, hemangioblastomas, and rhabdoid tumors are the types of brain tumors that are mostly in adults. Some types of brain tumors are more common in children than in adults. The most common types of pediatric tumors are medulloblastomas, low-grade astrocytomas (pilocytic), ependymomas, craniopharyngiomas, and brainstem gliomas. Primary brain tumors, malignant gliomas, pilocytic astrocytomas, and embryonal tumors are the most prevalent brain tumors in children, while malignant gliomas, pituitary tumors, and meningiomas are the most common brain tumors in adults [4]. Adult meningiomas are the most prevalent brain tumors, accounting for about 36 percent of all brain tumors in the United States Central Brain Tumor Registry (CBTRUS) [5]. According to the CBTRUS, an expected 24,000 new meningioma cases were diagnosed in the United States in 2015, with a 7.61 per 100,000 incidence rate. Because the lesions are typically asymptomatic and diagnosed by chance, it is very difficult to determine the prevalence. A prevalence from 1 percent to 2.7 percent has been found in several large autopsy investigations conducted before the introduction of MRI (magnetic resonance imaging) [6,7]. In one large autopsy study, 85 percent of the patients were detected by chance, while 15 percent were symptomatic. Because of the youngsters investigated and the threshold for the detection of tiny lesions on the imaging, the prevalence estimate from 0.3 percent to 0.9 percent in the present era of MRI is slightly lower [8,9]. Old diagnostic methods, such as MRI (magnetic resonance imaging), radiotherapy, and surgical resection, have demonstrated several limitations in clinical trials that are allied to their imaging sensitivity and therapeutic efficacy. The development of most of the advanced diagnostic and imaging approaches, such as functional MRI, positron emission tomography (PET), and computed tomography (CT), has made the early detection and treatment of brain cancer easier and more accurate [10]. MRI creates detailed images of the organs and tissues using a magnetic field and computer-generated radio waves. PET is an imaging technique that uses radioactive substances to visualize and measure changes in the metabolic processes, and in other physiological activities, including the blood flow, regional chemical composition, and absorption. CT is an imaging technique that is used to obtain detailed internal images of the body. Meningiomas are as frequent in females as in males, and they are 20% more prevalent in Black populations than in White populations [3,11]. The generality of meningiomas is benign (grade I), with from 5% to 20% (grade II) of them being atypical, and from 1% to 3% (grade III) being cancerous. Although benign meningiomas are a lesser cause of death, skull-based tumors can cause severe morbidity. Atypical and malignant meningiomas are linked to high rates of recurrence and considerable morbidity and mortality. Atypical meningiomas return about half of the time over a decade, and late recurrences are prevalent, with the majority of malignant meningiomas repeating at a median interval of two years [12]. Lung, breast, colorectal, melanoma, prostate, and renal cancers can cause brain metastases; however, breast cancer is primarily the result (around 70 percent) of brain metastases [13,14]. Radio chemotherapy is the current treatment for gliomas after surgical removal. As per the estimates of the National Cancer Institute of Canada in 2004, during the phase III trial of temozolomide adjuvant chemotherapy, it was found that the temozolomide trial showed a 27.2% survival rate, compared with glioblastoma patients treated with radiotherapy after surgery, and showed only a 10.9 percent survival rate [15,16]. There is no specific treatment for the recurrence of the illness, and patients are treated with investigational medications that are still in clinical studies [16]. For brain metastasis tumors, the available treatment options are surgery, radiation therapy, chemotherapy, targeted drug treatments, and immunotherapy. Brown et al. randomized 213 patients with 1–3 brain metastases to undergo treatment using stereotactic irradiation (STI) alone, or STI + whole-brain radiotherapy (WBRT) [17]. The combined therapy did not improve the overall survival (OS) rate. Goldberg et al. evaluated the efficacy of pembrolizumab, an anti-programmed cell death-1 antibody, for brain metastases in a phase II trial, and they reported that 29.7% of the patients with programmed cell death-ligand 1 (PD-L1)-positive NSCLC brain metastases responded to the treatment [18]. Oligodendroglioma emerges from oligodendrocytes, which create myelin and account for roughly 4% of the primary CNS tumors in babies, and 9% of the tumors in adults. Ependymomas, which arise from the brain’s ependymal cells, ventricles, and spinal cord, and are made up of cerebrospinal fluid (CSF), account for roughly 8–10 percent of the CNS malignancies in children [19].

All malignancies have an age-standardized incidence rate (ASIR) of 88.7 per 100,000 people in Saudi Arabia, while the age-standardized mortality rate (ASMR) in Saudi Arabia is 43.3 per 100,000 people [20]. Globocon 2020 reported that, in all Gulf nations, the greatest numbers of new cancer cases and deaths due to brain tumor have been found to be highest in Iraq, followed by Saudi Arabia, and they are the lowest in Qatar (Table 1) [21]. As per the Saudi Cancer Registry, the incidences of brain cancers in Saudi Arabian inhabitants are relatively low. It is estimated that brain tumors are one of the tenth most prevalent tumors in the Gulf Cooperation Council (GCC) countries, and the average age-standardized prevalence (ASR) for brain cancer was found to be 2.4 and 1.6 for males and females per 100,000 population, respectively. The largest nation in the Middle East, with an approximate population of around 22 million, is Saudi Arabia. According to the recent Saudi Cancer Registry study, executed in 2016, more than 300 new brain cancer cases accounted for 2.8% of the total cancer patients. The Riyadh region, followed by the Jouf region, the Northern region, the Eastern region, and the Qassim region were the five regions with the highest ASRs for brain cancer, whereas after the Jazan, Hail, and Asir areas, the Madina region had the lowest ASR for brain cancer [22,23].

To analyze the brain tumor distribution and trends in Saudi Arabia, we looked at the epidemiologic characteristics of ASIRs and CIRs, adjusted by region, year of diagnosis of brain tumor, age, diagnosis year, and the administrative districts, in Saudi Arabian inhabitants. The ASIR and CIR provide the exact data of the cancer incidences in a specific year in the population category of interest. An observational and thorough epidemiologic analysis of the brain tumor case distribution in the SCR from 2006 to 2016 was carried out to achieve this goal.

## 2. Materials and Methods

A retrospective observational cohort-based study of the incidence of mortality due to brain tumors was conducted on Saudi inhabitants, with the figures and data derived from the Saudi Cancer Registry (SCR) of the National Health Information Centre [24]. Annual cancer reports from the SCR provide information on the diagnosed cases of brain tumors in all locations in Saudi Arabia from 2006 to 2016. As a result, because the data have been taken from the open-access public domain, available through SCR annual reports, the study did not require ethical approval. We obtained detailed information on the cancer incidence ASIRs and CIRs for 13 administrative regions of Saudi Arabia, the diagnosis year, and sex. The current study followed the inclusion and exclusion criteria, it solely includes Saudi Arabian residents (citizens of Saudi Arabia), and it excludes non-Saudis (noncitizens). Based on these data, from 2006 to 2016, the present study was conducted using such reports to critically gather all information from the SCR to investigate the descriptive epidemiology of brain cancer among Saudi Arabian inhabitants.

We used SPSS version 20.0 (IBM Corporation, Armonk, NY, USA) and GraphPad Prism for the statistical analysis. In 13 regions of Saudi Arabia, the ASIRs and CIRs from the SCR reports were recorded from 2006 to 2016, and the differences between them were calculated to identify the brain cancer trends among men and women. An independent-sample *t*-test was performed for comparing the ASIR and CIR incidences of brain cancer among females and males. A nonparametric Kruskal–Wallis H test was also conducted for correlating the ASIRs and CIRs between the different administrative regions of Saudi Arabia. Additionally, the male/female ratio of brain cancer was also calculated from the age-specific incidence rate (AIR), ASIR, and CIR, categorized by year of diagnosis, age groups, and administrative regions. The overall percentage of the stage distribution of brain cancer from 2006 to 2016 was also identified among males and females.

## 3. Results

### 3.1. Brain Tumors among Males

**Figure 1:**
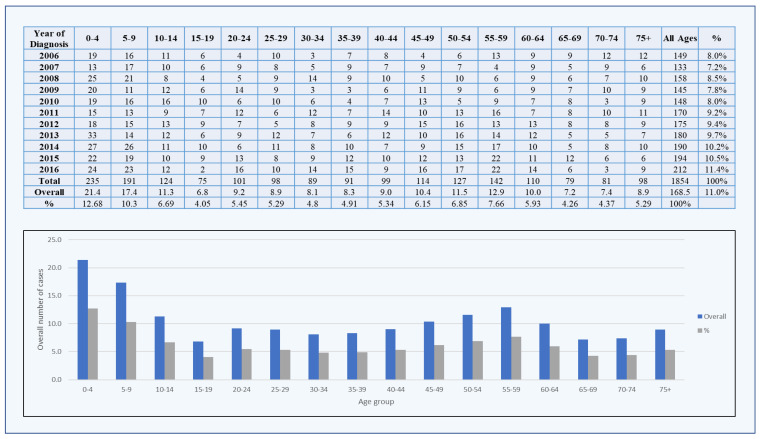
Numbers and percentages of brain tumors in males from 2006–2016.

In between 2006 and 2016, 1854 cases of male brain tumors were documented in the Saudi Cancer Registry [18]. The number of incidences of brain tumors climbed marginally (Table 2). The number of brain cancer cases in 2006 were 149, and in 2016, 212 cases were documented (Figure 1). According to the Saudi Cancer Registry, the highest number of brain cancer cases among the male population (212) was discovered in 2016. The proportion and mean number of reported brain tumor cases in Saudi Arabia were calculated by different age groups. The age-group class width was preserved at five years, commencing with 0–4 years, then 5–9 years, and ending with 70–74 years, with the last age group being more than 75 years. From 2006 to 2016, the annual percentage and mean of the male brain tumor cases were 11 percent and 168.54, respectively. Between 2006 and 2016, the majority of brain tumor cases were diagnosed in men and in children aged 0–4 years. Only children aged 0–4 years (235) were found to have the largest number of cases out of all 1854 instances. Children aged 5–9 years were followed by children aged 10–14 years (191). The age group 65–74 years had the lowest percentage and mean number of brain tumor cases (Figure 1).

**Figure 2:**
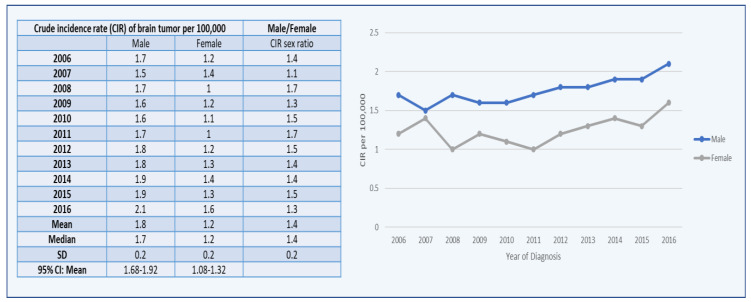
CIRs of brain tumor cases per 100,000 between male and female populations from 2006 to 2016.

The CIRs of the brain tumor incidents in Saudi Arabian men show a spike from 2006 to 2016, with a modest decrease from 2007 to 2010. (Figure 2). With a score of 2.1, the highest CIR was recorded in 2016, followed by 1.9 in 2015 and 2014. From 2006 to 2016, the average CIR sex ratio (male/female) was 1.8 (Figure 2).

The average CIRs of male brain tumors were categorized by thirteen regions in Saudi Arabia, as characterized in the SCR. Males in Riyadh had the highest mean CIR (2.5), followed by Makkah (1.8), and Eastern Province (1.8). The Kruskal–Wallis H test was used on the overall CIRs for all areas in males from 2006 to 2016 because the data did not follow a normal distribution or statistical significance for these locations in comparison with the other administrative areas.

**Figure 3:**
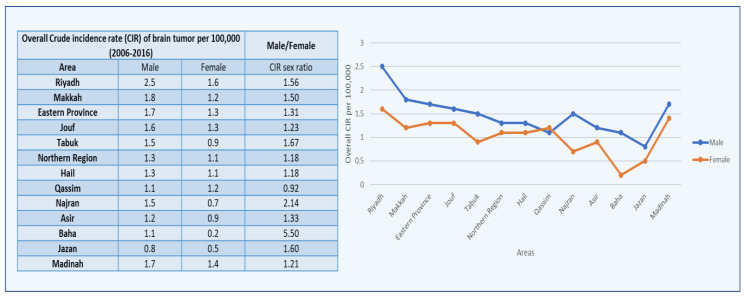
Overall CIRs of brain tumor cases per 100,000 between males and females according to administrative areas from 2006 to 2016.

**Figure 4:**
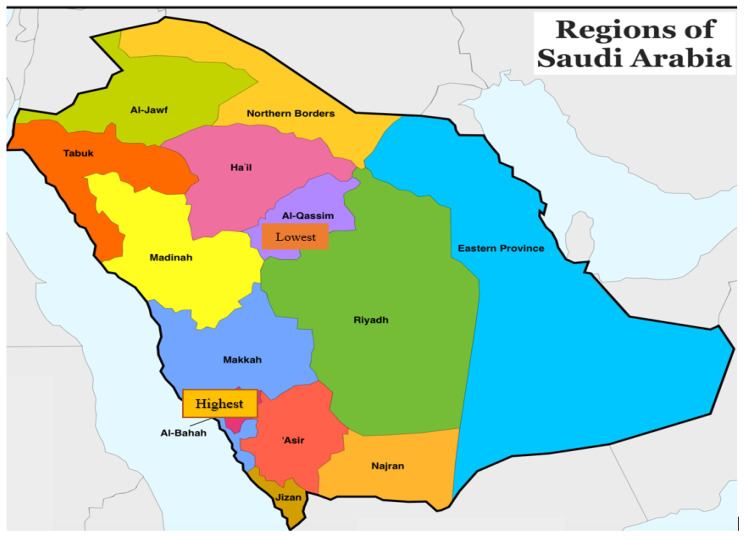
Brain Cancer: CIR by Geographical regions in Saudi Arabia. Source: https://www.orangesmile.com/travelguide/saudi-arabia/country-maps-provinces.htm.

Furthermore, the highest CIR sex ratio was 5.50, documented in the Baha Region of Saudi Arabia, followed by 2.14 in the Najran Region for brain tumors, while the lowest mean CIR was reported in the Jazan Region among males (Figure 3 and Figure 4).

**Figure 5:**
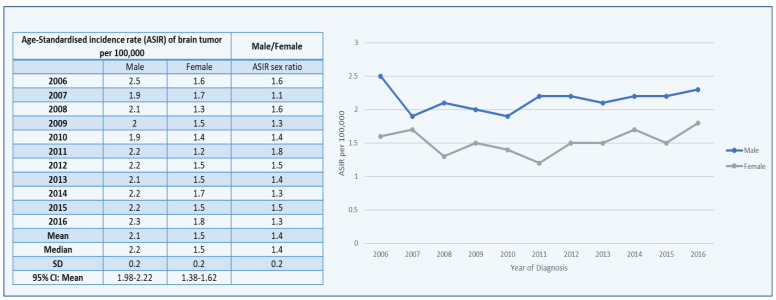
Age-standardized incidence rates (ASIRs) of brain tumor cases per 100,000 between males and females from 2006 to 2016.

The Saudi Cancer Registry revealed the age-standardized incidence rates (ASIRs) of brain tumors in males to year per 100,000 males (Figure 5). In Saudi Arabia, the value of the ASIRs for brain tumors remained nearly consistent from 2006 to 2016. The highest recorded value of the ASIR was 2.5 in 2006, followed by 2.3 in 2016. In the year 2010, the ASIR reached its lowest point of 1.9. Furthermore, among males with brain tumors, the mean standard deviation of the ASIR was 2.2 ± 0.2. From 2006 to 2016, the mean ± SD of the ASIR sex ratio per 100,000 people was 1.4 ± 0.2 (Figure 5).

**Figure 6:**
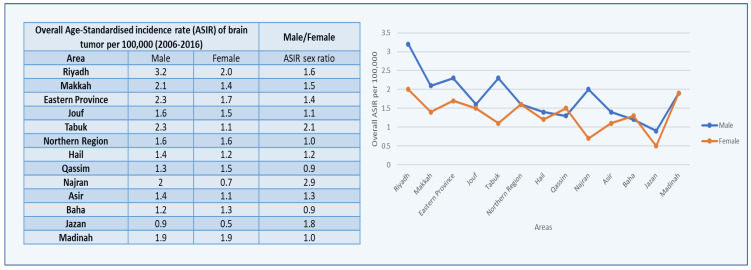
Overall ASIRs of brain tumor cases per 100,000 between males and females according to administrative areas from 2006 to 2016.

The average ASIRs of brain tumors in males were categorized by thirteen administrative areas in Saudi Arabia, according to the Saudi Cancer Registry. The highest mean ASIR was in the Riyadh region (3.2), followed by Eastern Province and Tabuk (2.3). As comparison to the data available for the administrative regions, some regions data do not follow normal distribution and statistical significance and, due to this, the Kruskal–Wallis H test was used on the overall ASIRs for all areas in males from 2006 to 2016. Furthermore, the highest ASIR sex ratio was found in Najran, Saudi Arabia, at 2.9, followed by 1.8 in Jazan for brain tumors, while the lowest mean ASIR was found in Jazan among men (Figure 6).

### 3.2. Brain Tumors among Females

**Figure 7:**
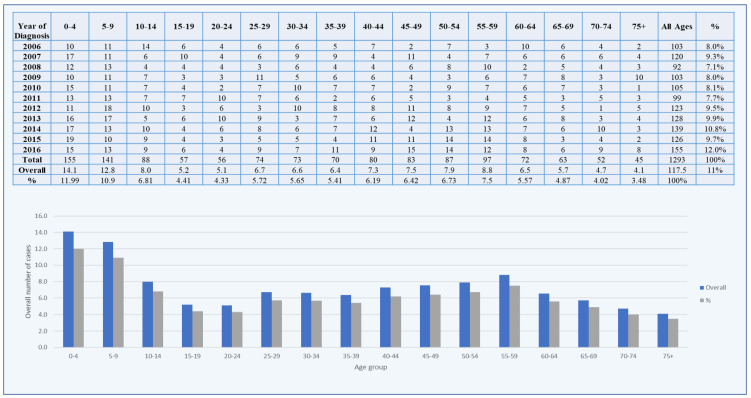
Numbers and percentages of brain tumors in females from 2006 to 2016.

A total of 1293 cases of female brain cancer between 2006 and 2016 were documented in Saudi Arabia [21]. Between 2006 and 2016, the number of instances of brain tumors climbed marginally. In 2006 and 2016, 103 and 155 cases of brain tumors were documented, respectively (Figure 7). According to the SCR, most of the cases of brain tumors among females (155) were discovered in 2016. The proportion and mean number of the reported brain tumor cases in Saudi Arabia were calculated by age group. From 2006 to 2016, the annual percentage and mean of the female brain tumor cases were 11 percent and 117.5, respectively. Between 2006 and 2016, the majority of the brain tumor cases were diagnosed in women and in children aged 0–4 years. Only children aged 0–4 years (235) were found to have the largest number of cases out of all 1854 instances. Children aged 5–9 years were followed by children aged 10–14 years (141). The age group 65–75 years and above had the lowest percentage and mean number of brain tumor cases (Figure 7).

The CIRs of the brain tumor cases in Saudi Arabian women increased from 2006 to 2016, with modest decreases in 2008 and 2010. With a score of 1.6, the highest CIR was recorded in 2016, followed by 1.4 in 2014 and 2007 (Figure 2).

The average CIRs of the female brain tumors were categorized by 13 administrative areas in Saudi Arabia, as characterized in the SCR. Females in Riyadh had the highest mean CIR (1.6), followed by Madinah (1.4), and then Jouf and Eastern Province (1.3). In comparison with the data available for the administrative regions, some regions’ data do not follow a normal distribution or statistical significance, and due to this, the Kruskal–Wallis H test was used on the overall CIRs for all areas in females from 2006 to 2016. Furthermore, the highest CIR sex ratio was 5.50 in the Baha region of Saudi Arabia, followed by 2.14 in the Najran region for brain tumors, while the lowest mean CIR was reported in the Baha region among females (Figure 3).

The Saudi Cancer Registry revealed the age-standardized incidence rates (ASIRs) of brain tumors in females to year per 100,000 females (Figure 5). In Saudi Arabia, the value of ASIRs for brain tumors remained nearly consistent from 2006 to 2016, with modest decreases in 2008 and 2011. The highest recorded value of the ASIR was 1.8 in 2016, followed by 1.7 in 2014 and 2007. In the year 2011, the ASIR reached its lowest point of 1.2. Furthermore, among females with brain tumors, the mean ± SD of the ASIR was 1.5 ± 0.2 (Figure 6).

The average ASIRs of brain tumors in females were categorized by thirteen administrative areas in Saudi Arabia, according to the Saudi Cancer Registry. The highest mean ASIR was in the Riyadh region (2.0), followed by Madinah (1.9), and Eastern Province (17). The lowest mean ASIR was found in Jazan among women (Figure 7).

## 4. Discussion

In Saudi Arabia, the most prevalent cancers are breast, colorectal, prostate, brain, lymphoma, kidney, and thyroid. Their prevalence rates and OR values (95% CI), respectively, are as follows: breast cancer: 53% and 0.93 (0.84–1.00); colon-rectal cancer (CRC): 50.9% and 1.2 (0.81–1.77); prostate cancer: 42.6% and 3.2 (0.88–31.11); brain/central nervous system cancer: 9.6% and 2.3 (0.01–4.2); Hodgkin’s and non-Hodgkin’s lymphoma: 9.2% and 3.02 (1.48–6.17); kidney cancer: 4.6% and 2.05 (1.61–2.61); thyroid cancer: 12.9% and 6.77 (2.34–19.53) [22].

For a better understanding of the disease in Saudi Arabia’s population, detailed analyses of the CIRs and ASIRs of the cases of brain tumors should be necessary throughout all the core areas of Saudi Arabia. The CIR and ASIR trends of brain tumors were examined in the current study. This research provides a thorough examination of the distribution of brain tumors among males and females in Saudi Arabia’s main administrative regions. The aim of the current study is to identify the CIR and ASIR patterns from 2006 to 2016 among the brain cancer cases in Saudi Arabia. It is one of the major descriptive epidemiological studies that has evaluated the spatial/temporal distribution of brain cancer among males and females from 2006 to 2016 in different regions of Saudi Arabia that is based on the PubMed database and Saudi Cancer Registry data. In this present study, the actual state of the brain cancer trend was examined, and the significance of brain cancer in the Saudi Arabia population was explored.

As per the study, the major suggestion is that there is an urgent need for advanced medical and surgical tools for the early detection of the disease, and the long-term management is perhaps the most significant global health concern associated with brain tumors. In the current time, we are in an advanced phase of technology, but at present, there is no simple population-wide screening test for brain tumors that allows for early and uniform identification. Moreover, symptoms such as headache or seizure are often too prevalent and nonspecific to indicate the need for additional radiological testing. Worldwide, headaches are the most prevalent form of neurological morbidity; however, only a small percentage of headache sufferers have brain tumors [23]. Before a conclusive radiological diagnosis, patients with brain tumors typically appear with a variety of nonspecific symptoms and signs that escalate to life-threatening circumstances. Advanced and expensive imaging techniques are required for the diagnosis and subsequent treatment planning, which are not widely available in many places. However, such technologies were becoming more extensively spread over the time period analyzed. Despite efforts to adjust for underascertainment, ascertainment bias may explain some of the increase in the brain tumor incidence during this time, but the extent to which this bias contributes to the overall increase in the age-standardized incidence rates deserves more research [24].

From 2006 to 2016, the Riyadh region had the highest mean ASIRs for brain tumors in both males and females, according to the current study. This shows that, in comparison with other places, the people of Riyadh are particularly sensitive to brain tumors. Furthermore, lifestyle, environment, and genetic variables may be linked to an increase in the ASIRs of brain tumors in both males and females in the Riyadh community. As a result, a comprehensive epidemiologic investigation is needed to decipher the important risk factors connected to the growth of ASIR brain tumors in Saudi Arabia’s Riyadh region. The presence of manufacturers and enterprises in the Riyadh region may be one of the major contributing factors in the increasing rate of incidence [25]. According to Feldman et al. (2014), vitamin-D deficiency raises the risk of cancer development, and taking vitamin-D supplements could be a safe and effective way to reduce the cancer incidence [26]. Vitamin-D deficiency affects around 84 percent of Saudi Arabia’s population [23]. Furthermore, most of the students at King Faisal University in Saudi Arabia have acute vitamin-D deficiency [27]. We also discovered that between 2006 and 2016, Jazan had the lowest mean ASIRs for brain tumors in both males and females. The low incidence rates in places with lower ASIRs could be attributed to differences in environmental conditions or other factors when compared with regions with higher ASIRs. The low incidence rates in certain locations may be due to a lack of adaption to Western lifestyles or businesses, as well as insufficient healthcare facilities and availability, as compared with regions with high incidence rates [28,29]. In comparison with Jazan, which had only a 2% obesity prevalence, the Riyadh region of Saudi Arabia had a 35% obesity prevalence, and a high incidence rate of brain tumors in both males and females [30]. This increase might be attributed to the change in the Saudi population lifestyle (adopting the Western model), a lack of cancer awareness, a lack of screening and early-detection programs, and social barriers toward cancer investigations. As a result, research into environmental, lifestyle, obesity, sedentary lifestyle, tobacco use, genetic variables, and iodine and Vitamin-D deficiency represent the apparent cancer risk factors in Saudi Arabia [31].

During the years 2006–2016, there were significant differences in the CIRs of brain tumors among males and females in various administrative districts (Figure 3). Males (3.2) and females (2.0) had the highest CIRs in the Riyadh region. In the Jazan region, the smallest significant change in the CIR was seen in both males and females. The maximum ASIR and CIR were found in the Riyadh region in both males and females. This region has the highest CIRs and ASIRs across males and females, indicating that it is at the top in the major significant increases in the CIRs and ASIRs for brain tumors from 2006 to 2016.

In a meta-analysis study, persons who ate meals high in nitrogenous components had a two-to-one odds ratio compared with those who did not [32]. Disease awareness, a healthy lifestyle, and a decrease in age for brain tumor screening would aid in the early detection and diagnosis of the disease, and it would be a promising approach to minimizing the incidence rates in the young and adult populations, which may cause problems for a country’s healthcare system in the coming decades [33,34]. To lower the rate of morbidity, mortality, and incidence of brain tumors, national and regional initiatives, strategies, and multilevel diagnoses are necessary [35].

The distribution of brain tumors in the Saudi Arabian population is described in depth. Over the last ten years, the number of people diagnosed with brain tumors has increased in Saudi Arabia. The findings of this descriptive epidemiological study may be helpful in formulating better public health policies with resource allocation and strategic planning on a national level for brain cancer in Saudi Arabia, and they may be valuable in making future hypotheses about the possible brain cancer risk factors in the most frequently affected regions by prospective epidemiological studies that identify the association between the disease and exposure.

## 5. Conclusions

The present study concluded that there was a rise in the crude incidence rates and age-standardized incidence rates for brain cancer among Saudi inhabitants. From 2006 to 2016, the Riyadh region had the highest mean ASIRs and CIRs for brain tumors in males and females, whereas the Jazan and Northern regions had the lowest mean ASIRs in males and females, respectively. To further understand the roles of the probable brain tumor variables in the Saudi Arabian population, more research is needed. The study should aid in generating brain-cancer-related epidemiological information about the Saudi Arabian population, and should ultimately help in understanding the cancer trends, which could help in the efforts to manage the incidence and mortality of cancer in the future, and in implementing a management, screening, and cancer prevention strategy in the country.

## Figures and Tables

**Table 1:** Assessed numbers of new cases and deaths in 2020 (per 100,000 individuals) in both sexes among Gulf nations [17].

Country	Both Sexes		Males		Females	
	New Cases	Deaths	New Cases	Deaths	New Cases	Deaths
Iraq	1600	1366	851	734	749	632
Saudi Arabia	598	486	425	347	173	139
UAE	119	98	73	62	46	36
Oman	113	93	87	70	26	23
Kuwait	68	57	51	45	17	12
Bahrain	40	32	24	18	16	14
Qatar	39	31	30	23	9	8

**Table 2:** The differences between ASIRs and CIRs of brain tumors between 2016 and 2006.

Area	Sex	ASIR/CIR	2016	2006	Difference
Riyadh	Male	ASIR	3.4	2.8	0.6
		CIR	3	2	1
	Female	ASIR	2.9	1.8	1.1
		CIR	2.4	1.3	1.1
Makkah	Male	ASIR	2.3	2.1	0.2
		CIR	2	1.6	0.4
	Female	ASIR	1.6	1.3	0.3
		CIR	1.5	1.1	0.4
Eastern Province	Male	ASIR	2.9	2.9	0
		CIR	2.4	1.8	0.6
	Female	ASIR	1.6	1.3	0.3
		CIR	1.3	1.1	0.2
Jouf	Male	ASIR	2.3	2.3	0
		CIR	2.1	1.8	0.3
	Female	ASIR	0.9	0.5	0.4
		CIR	0.5	0.6	−0.1
Tabuk	Male	ASIR	1.9	7.7	−5.8
		CIR	1.6	3.4	−1.8
	Female	ASIR	1.7	1.6	0.1
		CIR	1.4	1	0.4
Northern Region	Male	ASIR	1.1	2.7	−1.6
		CIR	1.4	2.4	−1
	Female	ASIR	0.6	0.6	0
		CIR	0.7	0.8	−0.1
Hail	Male	ASIR	0.8	1.1	−0.3
		CIR	0.8	1.3	−0.5
	Female	ASIR	1.2	2.8	−1.6
		CIR	1.1	2.5	−1.4
Qassim	Male	ASIR	1.5	2.3	−0.8
		CIR	1.4	1.6	−0.2
	Female	ASIR	1.1	0.6	0.5
		CIR	1	0.7	0.3
Najran	Male	ASIR	4	1.1	2.9
		CIR	3.7	0.5	3.2
	Female	ASIR	0.4	1.6	−1.2
		CIR	0.5	1.6	−1.1
Asir	Male	ASIR	1.2	1.7	−0.5
		CIR	1.2	1.2	0
	Female	ASIR	1.5	0.4	1.1
		CIR	1.4	0.5	0.9
Baha	Male	ASIR	1	0	1
		CIR	1.1	0	1.1
	Female	ASIR	1.1	1	0.1
		CIR	1.1	0.6	0.5
Jazan	Male	ASIR	0.3	1.3	−1
		CIR	0.3	1.2	−0.9
	Female	ASIR	0.6	0.6	0
		CIR	0.5	0.4	0.1
Madinah	Male	ASIR	1.7	1.9	−0.2
		CIR	1.6	1.9	−0.3
	Female	ASIR	2.5	4.9	−2.4
		CIR	2.2	2.8	−0.6

## Data Availability

The data used to support the findings of this study are included within the article.
